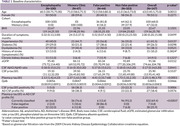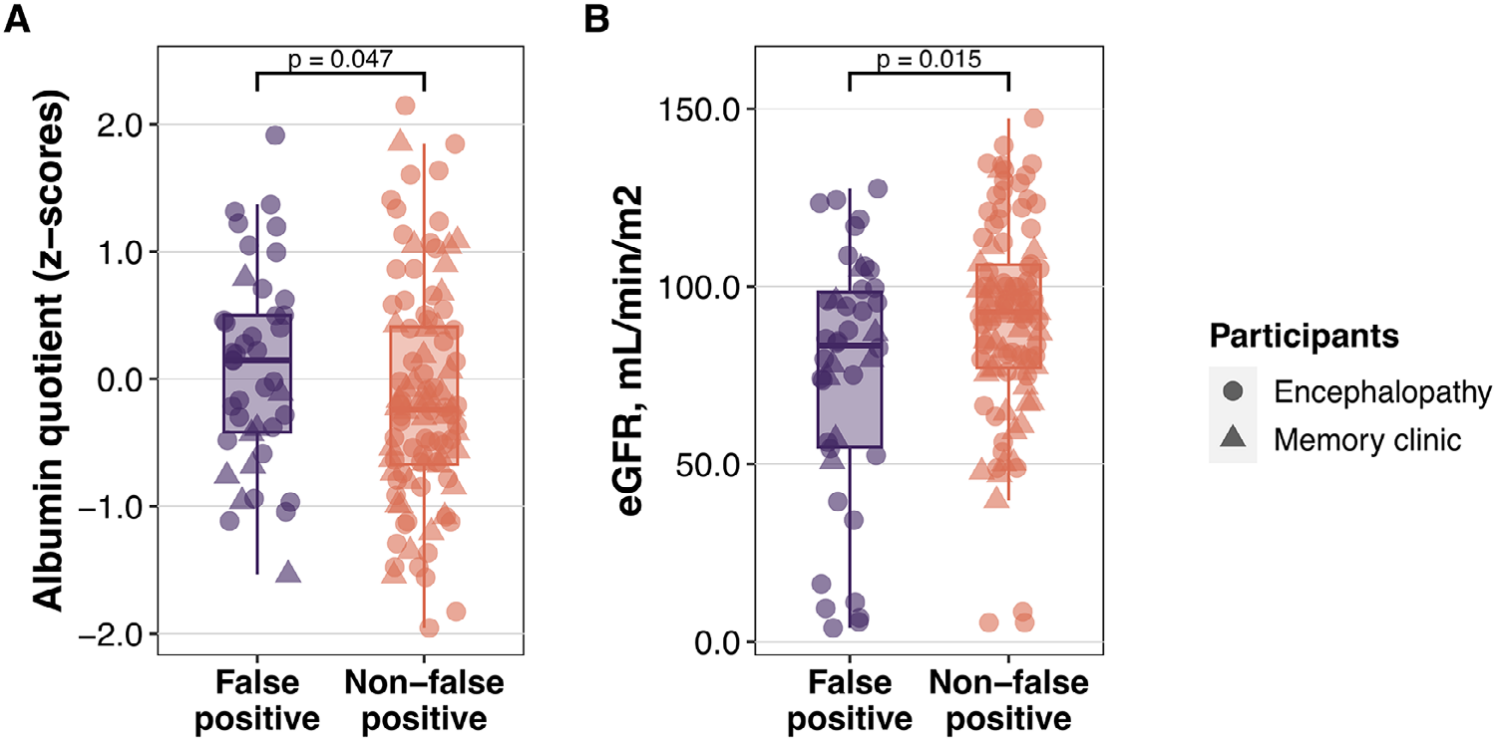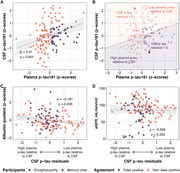# The effects of blood‐brain barrier permeability on Alzheimer’s disease plasma biomarkers and their impact on clinical performance

**DOI:** 10.1002/alz.091208

**Published:** 2025-01-09

**Authors:** Nattanich Pornteparak, Poosanu Thanapornsangsuth, Thanakit Pongpitakmetha, Tatchaporn Ongphichetmetha, Juthamas Rianaree, Watayuth Luechaipanit, Thanaporn Haethaisong, Adipa Chongsuksantikul, Yuttachai Likitjaroen, Thiravat Hemachudha

**Affiliations:** ^1^ Faculty of Medicine, Chulalongkorn University, Bangkok Thailand; ^2^ Department of Medicine, Banphaeo General Hospital, Samutsakhon Thailand; ^3^ Thai Red Cross Emerging Infectious Diseases Health Science Centre, King Chulalongkorn Memorial Hospital, Bangkok Thailand; ^4^ Memory Clinic, King Chulalongkorn Memorial Hospital, Bangkok Thailand; ^5^ Chula Neuroscience Center, King Chulalongkorn Memorial Hospital, Bangkok Thailand; ^6^ Siriraj Neuroimmunology Center, Faculty of Medicine Siriraj Hospital, Mahidol University, Bangkok Thailand; ^7^ Neurocognitive Unit, Division of Neurology, Faculty of Medicine, Chulalongkorn University, Bangkok Thailand; ^8^ Division of Neurology, Department of Medicine, Faculty of Medicine, Chulalongkorn University, Bangkok Thailand

## Abstract

**Background:**

The validation of blood‐based biomarkers presents a promising role in Alzheimer's disease (AD) diagnosis owing to their accessibility and diminished invasiveness. However, despite awareness of confounding factors like kidney function, inaccuracies persist in AD diagnosis using plasma p‐tau. Notably, diverse conditions that modify blood‐brain barrier (BBB) permeability have been linked to high plasma p‐tau levels, irrespective of AD pathophysiology.

**Methods:**

Data were sourced from pooling participants from preceding studies on individuals with encephalopathy and from patients attending a memory clinic, where both plasma and cerebrospinal fluid (CSF) were acquired. Stratifying participants based on binary plasma and CSF p‐tau181 results (using independently validated cut‐offs), the study compared the CSF/plasma albumin quotient (qAlb) between false positive and non‐false positive groups. Univariable linear regression predicting CSF p‐tau181 from plasma p‐tau181 was employed, facilitating the quantification of CSF p‐tau residuals for each participant. The correlations between residuals and qAlb as well as residuals and kidney were examined. A multivariable logistic regression model incorporating qAlb and plasma p‐tau181 to predict AD CSF profile was developed using encephalopathy participants and tested on the memory clinic patients.

**Results:**

A total of 147 participants were included. The qAlb is significantly elevated in the plasma p‐tau false positive group (0.0066 vs. 0.0049, p=0.04658). A notable negative correlation exists between qAlb and CSF p‐tau residuals (Spearman rho = ‐0.0986, p = 0.0347), while no correlation was observed between kidney function and CSF p‐tau residuals (Spearman rho = ‐0.1806, p = 0.2515). Plasma p‐tau181 and qAlb predict AD in the memory clinic with the AUC of 0.78 (95% CI 0.64‐0.92), not different from using p‐tau alone (AUC 0.7778 vs 0.7759, p=0.8626).

**Conclusion:**

BBB permeability influences the prediction of CSF p‐tau181 from plasma p‐tau181, notably leading to excessive level of plasma p‐tau (ie. false positives). Conversely, in evaluating the effectiveness of integrating qAlb with plasma p‐tau181 for the prediction of AD CSF profiles in clinical contexts, no difference from plasma p‐tau alone was observed. Future investigations should explore whether other biomarkers of BBB influence plasma biomarkers in a similar direction and are useful in identifying potential incorrectly classified patients.